# Biophysical characterization of low-frequency ultrasound interaction with dental pulp stem cells

**DOI:** 10.1186/2050-5736-1-12

**Published:** 2013-08-01

**Authors:** Sleiman R Ghorayeb, Upen S Patel, A Damien Walmsley, Ben A Scheven

**Affiliations:** 1 School of Engineering and Applied Sciences, Ultrasound Research Laboratory, Hofstra University, 104 Weed Hall, Hempstead, NY 11549, USA; 2 Orthopaedics Research Laboratory, FIMR, North Shore Hospital, Manhassett, NY 11030, USA; 3 School of Dentistry, College of Medical and Dental Sciences, University of Birmingham, St Chad's Queensway, Birmingham B4 6NN, UK

**Keywords:** Therapeutic ultrasound, Kilohertz-range ultrasound, Dental tissues, Tooth, Dental pulp, Tissue repair and regeneration, Dentine repair, Finite element modelling

## Abstract

**Background:**

Low-intensity ultrasound is considered an effective non-invasive therapy to stimulate hard tissue repair, in particular to accelerate delayed non-union bone fracture healing. More recently, ultrasound has been proposed as a therapeutic tool to repair and regenerate dental tissues. Our recent work suggested that low-frequency kilohertz-range ultrasound is able to interact with dental pulp cells which could have potential to stimulate dentine reparative processes and hence promote the viability and longevity of teeth.

**Methods:**

In this study, the biophysical characteristics of low-frequency ultrasound transmission through teeth towards the dental pulp were explored. We conducted cell culture studies using an odontoblast-like/dental pulp cell line, MDPC-23. Half of the samples underwent ultrasound exposure while the other half underwent ‘sham treatment’ where the transducer was submerged into the medium but no ultrasound was generated. Ultrasound was applied directly to the cell cultures using a therapeutic ultrasound device at a frequency of 45 kHz with intensity settings of 10, 25 and 75 mW/cm^2^ for 5 min. Following ultrasound treatment, the odontoblast-like cells were detached from the culture using a 0.25% Trypsin/EDTA solution, and viable cell numbers were counted. Two-dimensional tooth models based on μ-CT 2D images of the teeth were analyzed using COMSOL as the finite element analysis platform. This was used to confirm experimental results and to demonstrate the potential theory that with the correct combination of frequency and intensity, a tooth can be repaired using small doses of ultrasound. Frequencies in the 30 kHz–1 MHz range were analyzed. For each frequency, pressure/intensity plots provided information on how the intensity changes at each point throughout the propagation path. Spatial peak temporal average (SPTA) intensity was calculated and related to existing optimal spatial average temporal average (SATA) intensity deemed effective for cell proliferation during tooth repair.

**Results:**

The results demonstrate that odontoblast MDPC-23 cell numbers were significantly increased following three consecutive ultrasound treatments over a 7-day culture period as compared with sham controls underscoring the anabolic effects of ultrasound on these cells. Data show a distinct increase in cell number compared to the sham data after ultrasound treatment for intensities of 10 and 25 mW/cm^2^ (*p* < 0.05 and *p* < 0.01, respectively). Using finite element analysis, we demonstrated that ultrasound does indeed propagate through the mineralized layers of the teeth and into the pulp chamber where it forms a ‘therapeutic’ force field to interact with the living dental pulp cells. This allowed us to observe the pressure/intensity of the wave as it propagates throughout the tooth. A selection of time-dependent snapshots of the pressure/intensity reveal that the lower frequency waves propagate to the pulp and remain within the chamber for a while, which is ideal for cell excitation. Input frequencies and pressures of 30 kHz (70 Pa) and 45 kHz (31 kPa), respectively, with an average SPTA of up to 120 mW/cm^2^ in the pulp seem to be optimal and agree with the SATA intensities reported experimentally.

**Conclusions:**

Our data suggest that ultrasound can be harnessed to propagate to the dental pulp region where it can interact with the living cells to promote dentine repair. Further research is required to analyze the precise physical and biological interactions of low-frequency ultrasound with the dental pulp to develop a novel non-invasive tool for dental tissue regeneration.

## Background

Ultrasound has various industrial and medical applications. In medical imaging, it is recognized as an important and useful clinical tool in (prenatal) screening, diagnostics and surgery. In dentistry, ultrasound use is mostly limited to oral surface cleaning (i.e. removal of plaque and calculus) or root canal treatment [1]. Studies have also evaluated the use of high-frequency imaging ultrasound for dental diagnostic purposes as reported by Ghorayeb et al. [2,3].

Therapeutic ultrasound for healing and repair of tissues following injury or disease is increasingly gaining interest in the scientific and clinical community. In particular, the notion that low-intensity pulsed ultrasound is an effective tool to accelerate bone fracture healing highlights the exciting potential of ultrasound in hard tissue repair and engineering [4-6]. This paper addresses the question as to whether ultrasound can be used as a non-invasive biomechanical therapy to promote dental health and tissue repair. Oral health is essential for human health and well being, whilst dental disease affects the quality of life for individuals worldwide imposing an immense burden on healthcare systems as reported by the World Health Organization [7]. Despite advances in restorative materials, traditional dental treatments using filling materials are relatively inefficient with approximately 50% of cases requiring revision within 5–10 years after treatment [8].

Teeth are living biomechanical tissues consisting of mineralized dentine which is covered by highly mineralized and non-vital enamel. The dentine has a tubular structure containing fluid and cell processes from odontoblasts that are located around the periphery of the viable pulp chamber within the core of teeth. Odontoblasts are viable and active throughout life and are responsible for dentine production. Following mild tooth decay or injury, the activity of existing odontoblasts can be upregulated to produce reactionary dentine involving formation and repair of extracellular mineralized (inter- and intratubular) dentine matrix that serves to seal off and protect the viable dental pulp core. However, in the event of a major obnoxious damage resulting in (localized) death of odontoblasts, progenitor or stem cells residing in the dental pulp can be activated to differentiate to new odontoblasts which in turn produce new reparative dentine [9,10].

Accumulating evidence from pre-clinical studies has indicated that low-intensity pulsed ultrasound is capable of directly stimulating the cartilage and bone cells to accelerate bone repair and regeneration [5,6,11-13]. Our previous work demonstrated the significant potential of ultrasound to stimulate dental cells [10,14,15].

Finite element (FE) methods are widely used in the understanding of physical and mechanical behaviour in various structures. FE has been used to solve equations which govern ultrasonic wave propagation in teeth [16-18]. Ghorayeb and colleagues showed that inflammation of the pulp could be modelled by density changes in the pulpal area [19]. The particular novelty of this project proposal is the analysis of ‘therapeutic’ low-frequency ultrasound propagation and its interaction with odontoblasts and the dental pulp. Apart from the complex multilayered and anisotropic tissue structure, the challenge herein is the relative low frequency (up to 1 MHz) and thus long wave range of therapeutic low-intensity (kHz-range) ultrasound. Recently, 2D finite difference modelling was used to estimate low-frequency ultrasound propagation in bone-like samples [20]. In addition, modelling techniques have been shown to be effective for simulating ultrasound propagation and interaction with complex structures and materials, including bone and periodontal ligament [21-23]. These approaches allow high-fidelity simulations to be benchmarked directly to biological experiments and then used systematically to understand in detail the internal interactions of ultrasound with intricate tissue structures. In order to better understand low-frequency and low-intensity ultrasound propagation in teeth and its interaction with the vital dental pulp core, this study is aimed to generate and analyze 2D models of tooth using micro-computed tomography (μ-CT) and finite element modelling. In addition, in order to relate the computational modelling with biological effect, we investigated the direct effects of different intensities of low-frequency ultrasound on dental pulp cells using an odontoblast-like pulpal cell line.

## Methods

Scheven et al. [14,15] investigated the effects of low-frequency (kHz-range) ultrasound, generally used in dentistry for dental scaling, on odontoblast-like cells. These studies have shown that a single exposure of odontoblast-like cell lines to low-frequency ultrasound resulted in distinct effects on cell vitality and cell behaviour. Ultrasound was able to stimulate the expression of genes and production of growth factors such as transforming growth factor β1 (TGFβ1) and vascular endothelial growth factor (VEGF) believed to be important for odontoblast activity and dentine repair. These promising data highlight the significant potential for the exploitation of ultrasound in novel dental regenerative therapies. This notion is supported by previous observations by Olgart et al. [24] who described that treatment of teeth with low-frequency ultrasound stimulated pulpal blood flow. Taken together, we have postulated that exposure of teeth to ultrasound may trigger cellular responses in the dentine–pulp complex, thereby possibly stimulating odontoblast activity and/or activating pulp mesenchymal stem cells inducing tooth repair [10,15]. Little is known, however, about the underlying biophysical mechanisms of action of ultrasound in hard tissues. Moreover, the precise relationship of ultrasound intensity and frequency with biological effect is unclear.

All studies were approved by and in compliance with the guidelines set by the institutional review board committee at the School of Dentistry, College of Medical and Dental Sciences, University of Birmingham (Birmingham, UK) and at Hofstra University (Hempstead, NY, USA).

### Finite element modelling

This portion of the study addressed the characteristics of ultrasonic waves travelling through the different layers of mineralized dental tissues (enamel, dentine) and their interaction with the dentine–pulp complex using computational simulation modelling. Finite element was used to explore the potential theory that with the correct combination of frequency and intensity, a tooth can be repaired using small doses of ultrasound. In addition, the purposes of this study are (a) to accurately model the waves propagating in the teeth, (b) to observe safety issues by keeping track of the spatial peak temporal average (SPTA) intensity and (c) to relate this figure to existing optimal spatial average temporal average (SATA) intensity deemed effective for cell proliferation during tooth repair. It is envisaged that ultimately, these computational analyses will be related to biological effects and would facilitate the development of a dental therapeutic ultrasound device. The SPTA intensity (*I*_spta_) was calculated using the following relationship [17]:

(1) $$I_{\mathrm{spta}}=\frac{1}{T_{\mathrm{prf}}}\;\int \frac{p^{2}\left(0,F,t\right)}{\rho \;c_{o}}$$

where *T*_prf_ is the pulse repetition period (=1 ms), *ρ* is the density, *c*_o_ is the sound velocity in the tooth layers (Table 1) and *p*(0,*F*,*t*) is the ultrasonic pressure in the tooth calculated using the following:

(2) $$p\left(0,F,t\right)=\rho h_{o}\;\frac{\partial v\left(t\right)}{\partial t}$$

where *v*(*t*) is the velocity at the transducer–medium interface, and *h*_o_ is the depth at which the spatial peak intensity across the entire radiated ultrasonic beam is being measured. For a given focal length *F* and transducer disc radius *a*, *h*_o_ can be determined from the following:

(3) $$h_{o}=F-\sqrt{\left(F^{2}-a^{2}\right)}$$

**Table 1:** Densities and speed of sound in each tooth layer

**Material**	**Density (kg/m**^**3**^**)**	**Speed of sound (m/s)**
Enamel	3,000	6,250
Dentin	2,000	3,800
Pulp	1,000	1,570

The intent of this simulation was to see what combinations of frequencies and input pressures would yield a range of SPTA intensities between 30 and 120 mW/cm^2^ in the pulp region and then confirm that this level is maintained throughout the entire tooth in accordance with FDA regulations. Furthermore, this range (30–120 mW/cm^2^) was adopted as the absolute limit threshold at low ultrasonic frequencies that would stimulate bone repair and may also apply to tooth regeneration as determined using dental scalers [10,14,15].

**Figure 1:**
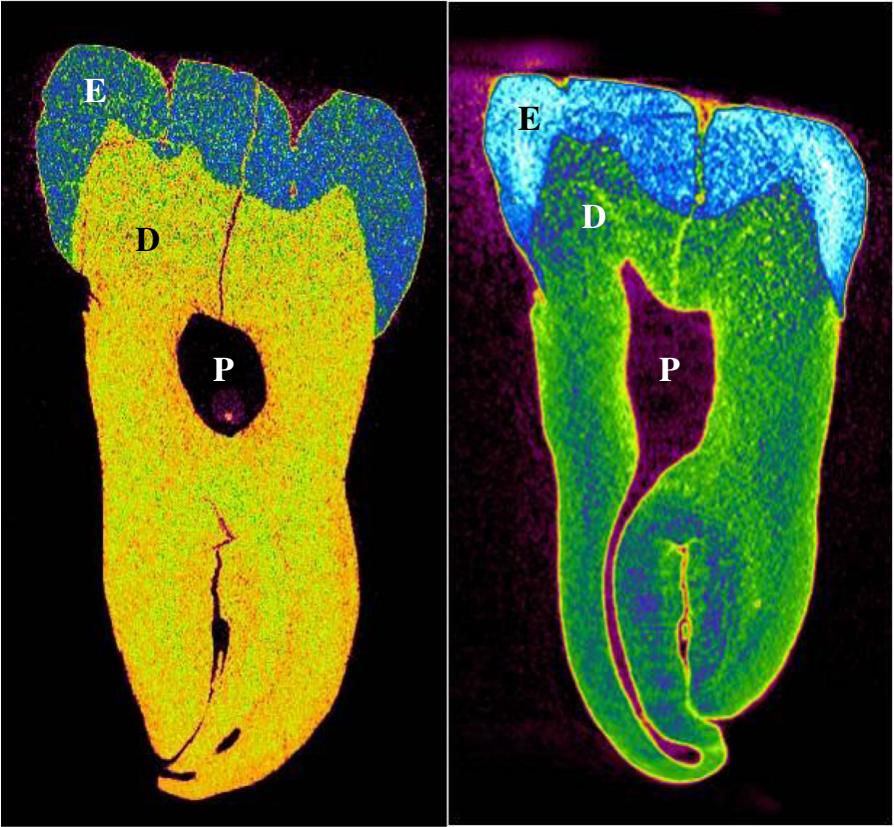
**μ-CT scans of two different teeth showing all three major layers including the pulp cavity.***E*, enamel; *D*, dentine; *P*, pulp.

Two-dimensional tooth models were analyzed using COMSOL (COMSOL, Inc., Burlington, MA, USA). Figure 1 shows typical μ-CT 2D images of the teeth. These were loaded into the acoustics module of COMSOL as the platform to be discretized and analyzed.

The input pressure pulse used is $\cos\;\left(2\mathrm{\pi ft}\right)\left(1-\cos\left(2\mathrm{\pi f}\frac{t}{3}\right)\right)\;$, where *f* is the acoustic frequency in Hertz, and *t* is time in seconds. Frequencies in the 30 kHz–1 MHz range were analyzed. For each frequency, the pressure/intensity plots provided information on how the intensity changes at each point throughout the propagation path.

### Cell cultures and ultrasound application

MDPC-23 is a proliferating cell line derived from fetal murine dental papilla expressing a range of odontoblast-like characteristics [14,15]. The MDPC-23 cells were cultured as an adherent monolayer in T75 flasks containing Dulbecco's modified Eagle Medium (DMEM) supplemented with 10% fetal bovine serum, 1% penicillin/streptomycin (Sigma-Aldrich®, Dorset, England, UK) and 200 mM glutamine (GlutaMAX™, Gibco®, Invitrogen™, Sigma-Aldrich®) in a humidified incubator with 5% carbon dioxide in air at 37°C. Near confluent cultures were detached from the culture plastic using Trypsin/EDTA (GIBCO, Paisley, UK) treatment, resuspended into a homogenous single cell suspension and seeded in three 6-well plates (Costar® tissue-culture treated, Corning®, Corning, NY, USA). On day zero, 50,000 cells were seeded in each well of the three 6-well plates and subsequently formed an adherent monolayer. The culture medium was replenished on days 1, 3, 5 and 7 with ultrasound treatment on days 2, 4 and 6. Each plate had three wells exposed to ultrasound and three control group that underwent ‘sham treatment’ where the transducer was submerged into the medium but no ultrasound was generated (0 mW/cm^2^). Ultrasound was applied directly to the cell cultures using a therapeutic ultrasound device (Duo Son, SRA Developments, Devon, UK) at a frequency of 45 kHz with intensity settings of 10, 25 and 75 mW/cm^2^ (SATA) for 5 min. On day 8 of culture, following ultrasound treatment, the odontoblast-like cells were detached from the culture using a 0.25% Trypsin/EDTA solution (Sigma-Aldrich®), and viable cell numbers were counted using a haemocytometer and trypan blue staining. Ultrasound intensity was calibrated using a hydrophone force field analyzer (SRA Developments, Devon, UK). Experimental temperature monitored using a digital thermometer (Iso-Tech IDM 207, Northants, UK) with a ‘K-type’ wire showed that the temperature of the culture medium only marginally increased by approximately 2–3°C after 30 min of ultrasound application indicating that thermal stress was negligible in the current experimental set-up.

## Results and discussion

### FE simulation results

**Figure 2:**
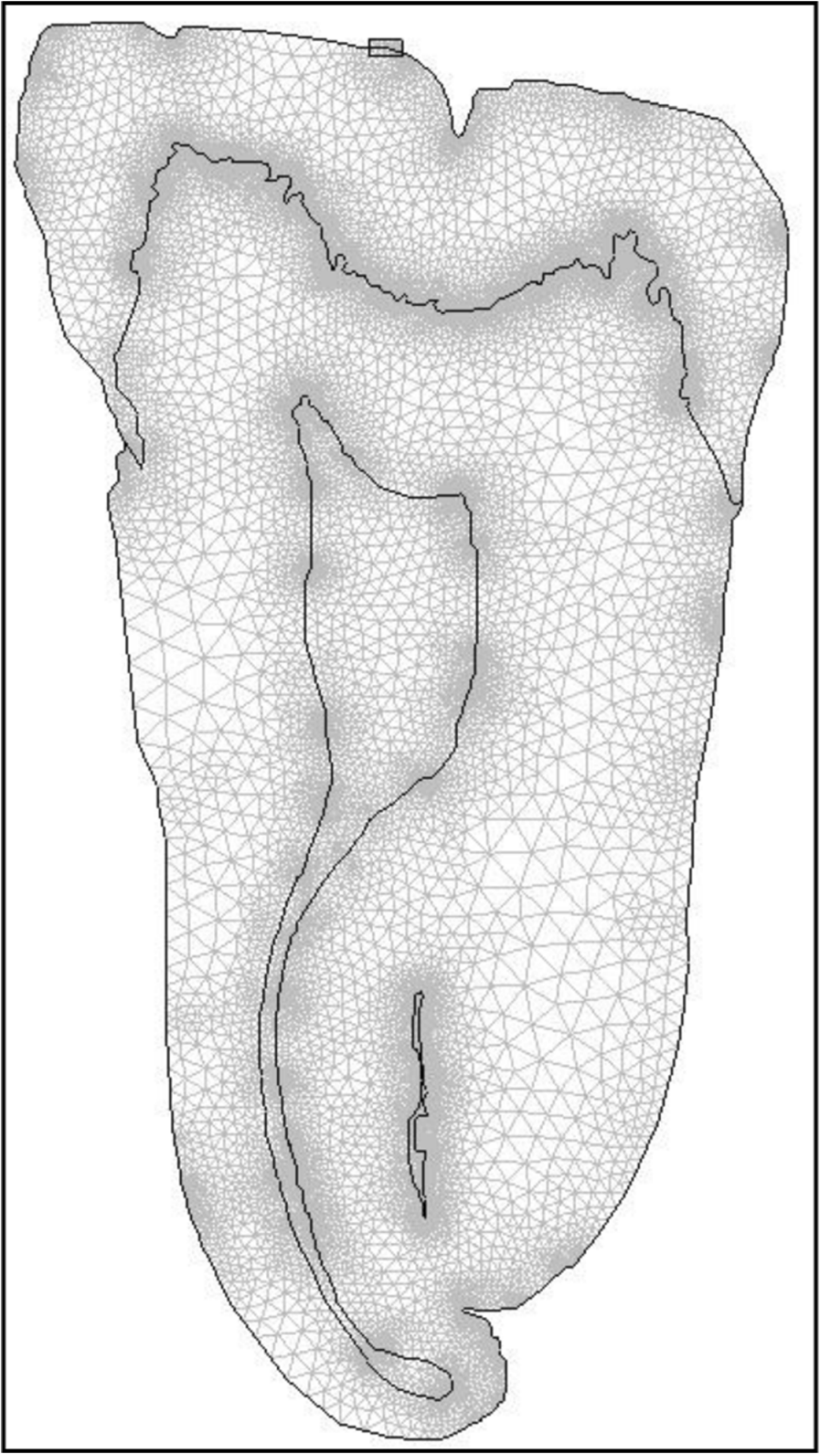
Discretization of one of the CT tooth scans.

Figure 2 shows the discretized tooth geometry.

**Figure 3:**
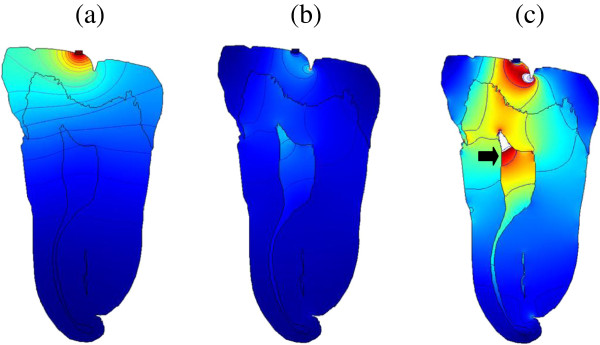
**Snapshots of pressure and intensity plots of (30 kHz, 70 Pa) ultrasonic wave throughout the tooth. (a)** Pressure at *t =* 1.5 μs, **(b)** intensity at *t =* 0.5 μs and **(c)** intensity at *t =* 3 μs. It shows high-intensity concentration in pulp (arrow) (Additional file 1).

**Figure 4:**
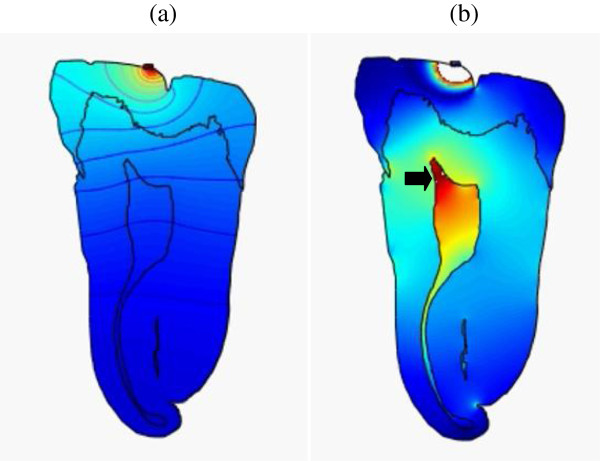
**Snapshots of pressure and intensity plots of (45 kHz, 31 kPa) ultrasonic wave throughout the tooth.(a)** Pressure at *t =* 1 μs and **(b)** intensity at *t =* 3.35 μs. It shows high-intensity concentration in pulp (arrow) (Additional file 2).

**Figure 5:**
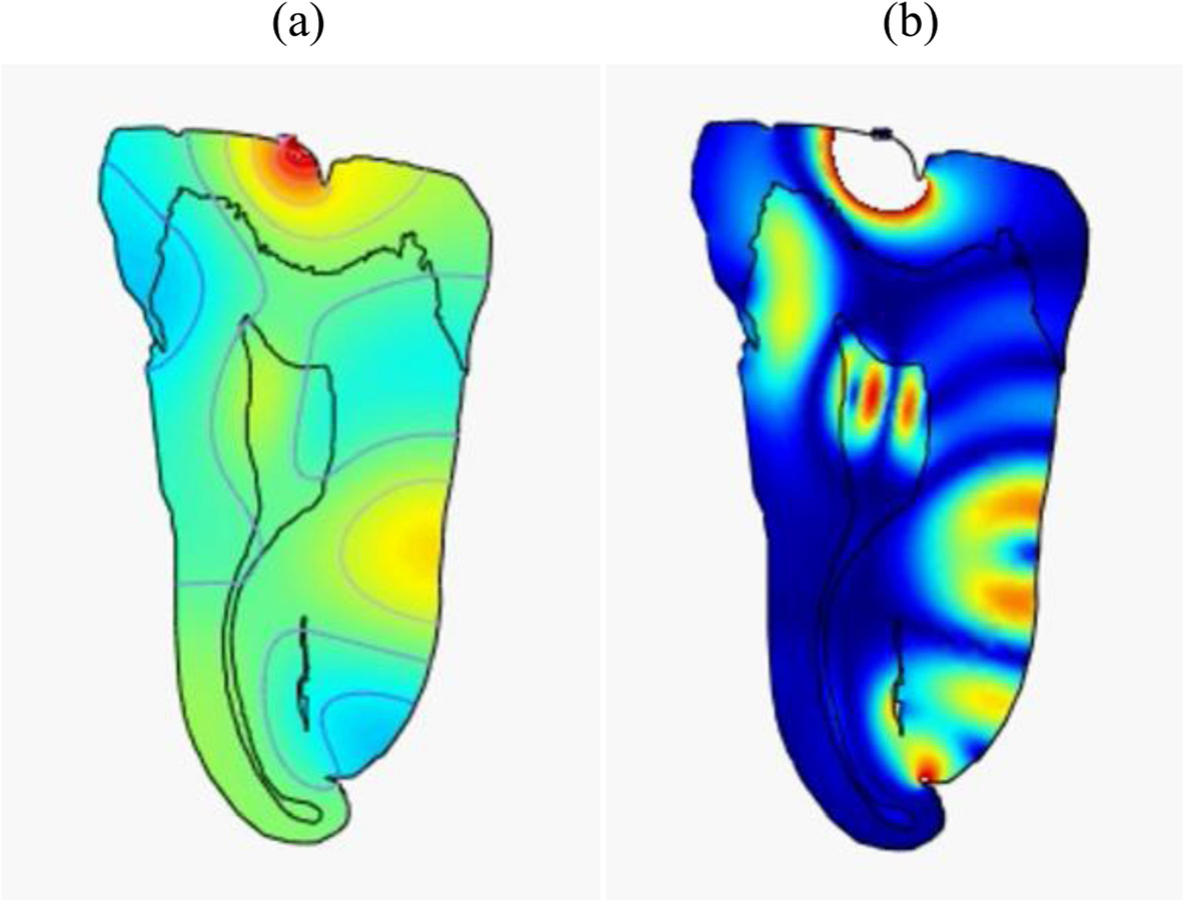
**Snapshots of pressure and intensity plots of (500 kHz, 200 kPa) ultrasonic wave throughout the tooth.(a)** Pressure at *t =* 1 μs and **(b)** intensity at *t =* 3 μs (Additional file 3).

**Figure 6:**
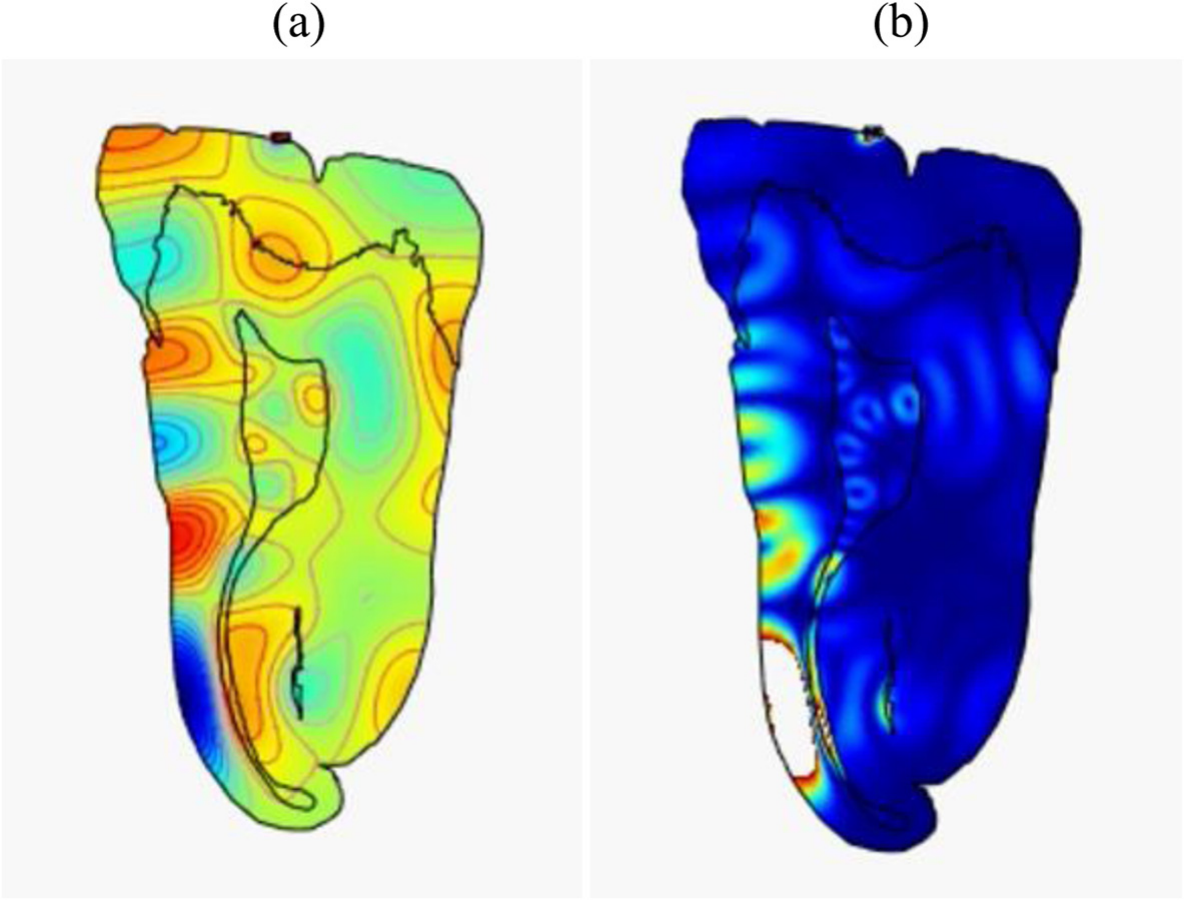
**Snapshots of pressure and intensity plots of (1 MHz, 19 kPa) ultrasonic wave throughout the tooth.(a)** Pressure at *t =* 1.5 μs and **(b)** intensity at *t =* 1.5 μs (Additional file 4).

Surface plots were superimposed over contour plots, and animations of these plots were created. This allowed us to see the pressure/intensity throughout the tooth while watching the wave propagate. The following results shown in Figures 3, 4, 5 and 6 (Additional files 1, 2, 3 and 4, respectively) exemplify a selection of time-dependent snapshots taken for frequencies in the 30 kHz–1 MHz range. For each frequency, the pressure/intensity plots are shown, providing information on how the intensity changes at each point throughout the propagation path.

The data as presented in Table 2 indicate the ‘best’ combinations of frequencies and input pressures that are within FDA regulations for ultrasonic exposure limits in the pulp. This means that using one of these combinations guarantees that the ultrasound intensity in the pulp will not exceed the maximum SPTA intensity of 120 mW/cm^2^ calculated using Equation 1.

**Table 2:** Upper limit for input pressures at the given frequencies

**Frequency (Hz)**	**Input pressure (Pa)**
30 k	70
45 k	43.5 k
50 k	127 k
55 k	85.5 k
60 k	109 k
65 k	149 k
70 k	139 k
75 k	340 k
80 k	200 k
85 k	400 k
90 k	210 k
95 k	231 k
100 k	170 k
125 k	59 k
150 k	162 k
175 k	214 k
200 k	135 k
225 k	80 k
250 k	221 k
275 k	149 k
300 k	300 k
500 k	90.1 k
600 k	25.65 k
700 k	331.6 k
800 k	84.88 k
900 k	15 k
1 M	55.7 k

**Figure 7:**
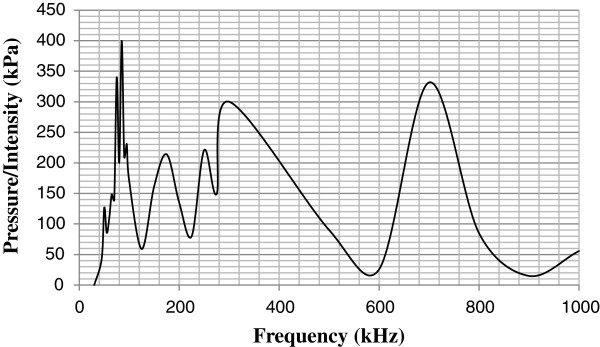
Plot showing relationship between ultrasonic wave pressure/intensity (in kPa) and frequency (in kHz).

Interestingly, when the pressures were plotted against their respective frequencies, there seemed to be no direct correlation that is valid for predicting more values, as seen in Figure 7.

Examining the results, it appears that the lower frequency waves propagate to the pulp and remain within the chamber for a while. This is optimal if considering the cells we want to excite are located there. The higher frequencies, due to the smaller wavelength, are more affected by imperfections in the boundaries, as reflected by the higher intensity surface waves that are distributed throughout the periphery of the tooth. Considering all of the results shown in Figures 3, 4, 5 and 6 (Additional files 1, 2, 3 and 4, respectively), the optimal input frequencies and pressures are the 30-kHz (70 Pa) and the 45-kHz (31 kPa) cases, respectively, as they generate an average of 120 mW/cm^2^ in the pulp. It is important to note that these frequencies correspond to standard dental scalers such as those of the Cavitron® scaler (DENTSPLY Professional, York, PA, USA) commonly used during dental hygiene and that Scheven et al. [14] and Olgart et al. [24] referred to in their studies. These cases can be seen as peak intensities (shown in red levels) in Figures 3 and 4 (Additional files 1 and 2). In these plots, any intensity over the limit of 120 mW/cm^2^ would not be displayed on this greyscale. This means that the combination of frequency and pressure in these figures is FDA safe. Also, the input pressure is considerably lower than all other frequencies tested, making it even more attractive and efficient. However, it should be emphasized that these results refer to the safe upper limits of the therapeutic dental application theories, but that further detailed studies are needed to clarify and thus confirm whether these regimens will provoke the specialization of dental pulp stem cells into odontoblast-like cells and therefore tooth regeneration.

### Low-frequency ultrasound stimulates MDPC-23 cell proliferation

**Figure 8:**
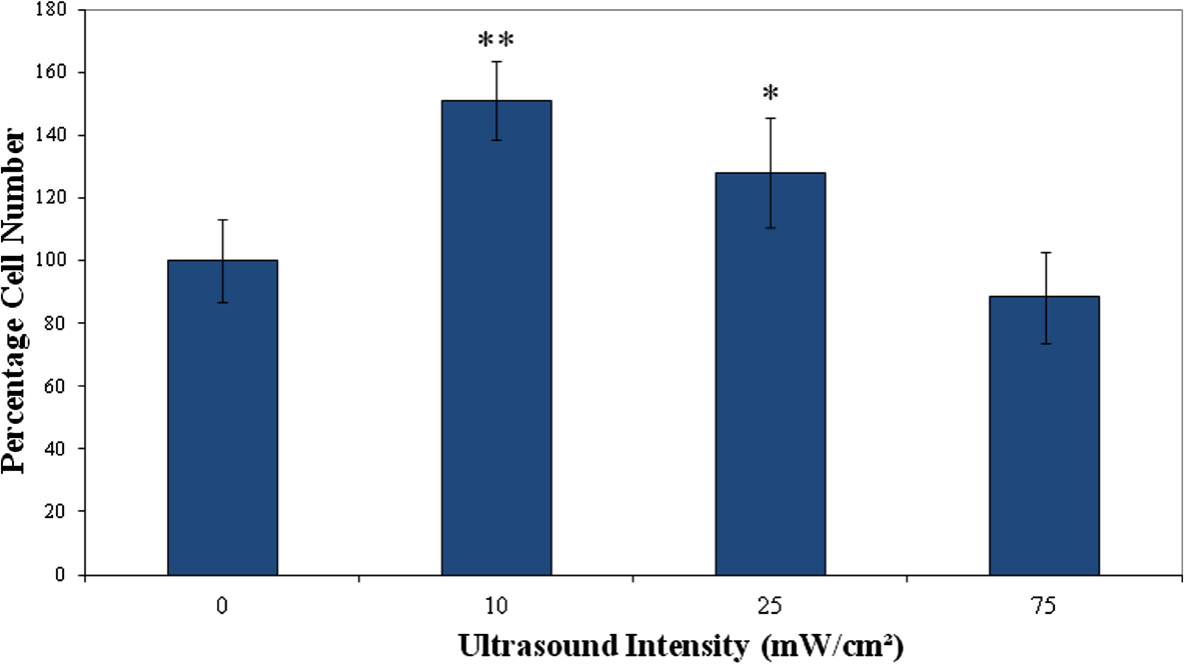
**MDPC-23 odontoblast-like percent change in cell numbers after treatment with low-frequency 45-kHz ultrasound.** The data are mean of three experiments using three replicates for each experiment. The graph shows the percentage of cell number compared to the control group. The error bars shown represent standard deviation values versus control values (^*^p < 0.05, ^**^p < 0.01).

Further to the above-described simulations, the next experiments were conducted to investigate the biological effects of low-frequency (kHz-range) ultrasound on odontoblast-like cells and whether these effects are dose-dependent using a calibrated therapeutic ultrasound device. The results demonstrate that odontoblast-like MDPC-23 cell numbers were significantly increased following three consecutive ultrasound treatments over a 7-day culture period as compared with sham controls. The graph shown in Figure 8 is a compilation of three data sets of three experiments, using three replicates for each experiment. The data show the percentage change in cell number compared to the sham data after ultrasound treatment for each ultrasound intensity (10, 25 and 75 mW/cm^2^). The error bars represent standard deviation values (*p* < 0.05 and *p* < 0.01) versus control values.

These findings indicate that ultrasound promoted cell proliferation at low-intensity therapeutic intensities. Ultrasound stimulation appeared to show a dose-dependent relationship with the greatest effect at intensities of 10 and 25 mW/cm^2^. Interestingly, these values correspond to the intensities generally used in low-intensity pulsed ultrasound treatment for bone fracture healing (25–30 mW/cm^2^) [5,6,25], although lower doses may be equally, if not, more effective in obtaining therapeutic effect. These findings also imply that biophysical stimulation by ultrasound may involve a threshold dose similar to the mechanostat threshold concept surrounding the theory of mechanical loading of bones [26,27]. However, there also seems an upper threshold where too high intensities may negate any positive biological/anabolic effect. These data linked with the computational modelling results shown earlier suggest that for safe and effective therapeutic use of dental ultrasound treatment, a relatively low-intensity application may deliver an efficient biological effect [28]. Our current findings were obtained from a 7-day culture experiment involving several consecutive ultrasound treatments.

Another point of clarification worth mentioning is that the SPTA and the SATA are related through the beam uniformity ratio (BUR). BUR is a quantitative indication of ultrasonic beam uniformity across the face of the transducer. A perfect ideal case is when BUR = 1. However, as uniformity worsens, BUR increases. The transducer used in our therapeutic device (Duo Son, SRA Developments, Ashburton South Devon, UK) produces a non-uniform diverging beam with an effective beam radiating area of 16.3 cm^2^ and a BUR ≅ 6. Spatial peak (SP) and spatial average (SA) figures are related by the following:

(4) $$\;\mathrm{SP}=\left(\mathrm{SA}\right)\left(\mathrm{BUR}\right)$$

Therefore, our ideal SATA figures of 10 and 25 mW/cm^2^ obtained in the experimental results convert to SPTA values of 60 and 150 mW/cm^2^, respectively, which overlap the range of SPTA intensity levels of 30–120 mW/cm^2^ calculated from the finite element analysis.

Of interest is that our recent work also indicated that a single dose of 25 mW/cm^2^ stimulated subsequent *in vitro* differentiation and mineralization [28]. These observations suggest that a single application of ultrasound could trigger cellular responses leading to proliferation and differentiation of odontoblasts. Further research is warranted to elucidate the clinical potential and biophysics of ultrasound within the dental pulp in order to harness and develop a suitable and efficient therapeutic tool for tooth repair.

## Conclusions

This study has modelled the transmission of low-intensity low-frequency ultrasound through the outer mineralized layers of the teeth to the dental pulp chamber. In addition, this research demonstrated that a single treatment of low-frequency ultrasound stimulates dental pulp cell proliferation using long-term MDPC-23 cell cultures. Thus, the data provide evidence that exposure of the teeth to low-frequency ultrasound may generate a therapeutic intensity within the dental pulp that may facilitate new reparative dentine formation.

## Competing interests

The authors declare that they have no competing interests.

## Authors’ contributions

SRG mentored the finite element modelling portion of this research project, provided guidelines and protocols for all simulation studies, analysis and interpretation of the data, wrote half of the manuscript and was responsible for the compilation of the complete manuscript and final submission. UP carried out the laboratory measurements and interpretation of the data. ADW contributed his expertise in ultrasound-based techniques as applied to dentistry. BAS mentored the *in vitro* study portion of this research project, provided clinical information about this application, participated in the interpretation of the data and wrote the sections pertaining to this aspect of the manuscript. All authors read and approved the final manuscript.

## Supplementary Material

Additional file 1 Snapshots of pressure and intensity plots of (30 kHz, 70 Pa) ultrasonic wave throughout the tooth. Click here for file

Additional file 2 Snapshots of pressure and intensity plots of (45 kHz, 31 kPa) ultrasonic wave throughout the tooth. Click here for file

Additional file 3 Snapshots of pressure and intensity plots of (500 kHz, 200 kPa) ultrasonic wave throughout the tooth. Click here for file

Additional file 4 Snapshots of pressure and intensity plots of (1 MHz, 19 kPa) ultrasonic wave throughout the tooth. Click here for file
